# Turnover of nurse anesthetists: the similarities and differences between countries

**DOI:** 10.1186/1472-6963-14-S2-P72

**Published:** 2014-07-07

**Authors:** Christine Mahoney, Joshua Lea, Irene Jillson, Vera Meeusen

**Affiliations:** 1 Management Dept, College of Business, Minnesota State University, Mankato, MN, USA; 2 School of Nursing and Health Studies, Georgetown University, Washington, DC, USA; 3 Catharina Hospital, Eindhoven, The Netherlands

## Background

For more than a decade, both the United States (US) and the Netherlands have been struggling to control health care costs. The demand for nurse anesthetists, or in the US, Certified Registered Nurse Anesthetists (CRNA), has increased in both countries due to the increased substitution of nurse anesthetists for anesthesiologists in surgery, with the goal of reducing health care costs. Additionally, the aging of the population in both countries has increased the demand for health care services. The health care systems and related outcomes of the two countries were quite dissimilar. The US relies more on private insurance than other high-income countries. Previously the Netherlands provided care for all citizens through non-competing regional sickness funds. These laws changed in 2006; moving the Netherlands’ system closer to the US system.

## Materials and methods

An online self-reporting questionnaire survey was performed among Dutch nurse anesthetists in 2007 and among US CRNAs in 2013. The questionnaires included validated scales to assess turnover intention, work climate, work context factors, burnout, job satisfaction, and personality dimensions.

## Results

The study of job retention of nurse anesthetists in The Netherlands found that. 1) burnout was predicted by personality traits and work climate; 2) job satisfaction was predicted by work climate and context, and 3) turnover intention was predicted by burnout and job satisfaction. The newer data collected from US CRNAs results mirror the findings on the Netherland’s data. For CRNAs in the US, burnout is predicted by both personality and work climate factors; job satisfaction is predicted by work climate and context factors, and turnover is predicted by both burnout and job satisfaction.

## Conclusions

These results suggest that the work climate and work context have the same impact on job satisfaction, and therefore on turnover intentions, across different health system types, different cultures, and different countries. Similarly, personality traits and work climate have the same impact on burnout, and therefore turnover intentions of nurse anesthetists. These findings make an important contribution because they suggest that healthcare management interventions to retain highly trained and skilled health professionals may be generalized across country boundaries and health system types.

